# Assessment of *Euphorbia hirta* L. Leaf, Flower, Stem and Root Extracts for Their Antibacterial and Antifungal Activity and Brine Shrimp Lethality

**DOI:** 10.3390/molecules15096008

**Published:** 2010-08-31

**Authors:** Mohammad Abu Basma Rajeh, Zakaria Zuraini, Sreenivasan Sasidharan, Lachimanan Yoga Latha, Santhanam Amutha

**Affiliations:** 1 School of Distance Education, Universiti Sains Malaysia, 11800, Pulau Pinang, Malaysia; E-Mail: zuraini@usm.my (Z.Z); 2 Institute for Research in Molecular Medicine (INFORMM), Universiti Sains Malaysia, 11800, Pulau Pinang, Malaysia; E-Mail: srisasidharan@yahoo.com (S.S); 3 School of Biotechnology, Madurai Kamaraj University, Madurai 625021, India

**Keywords:** antimicrobial, *Euphoribia hirta*, MIC, SEM, time-kill assay, *Artemia salina* napulii

## Abstract

The antimicrobial activities of the methanolic extracts of *Euphorbia hirta* Lleaves, flowers, stems and roots were evaluated against some medically important bacteria and yeast using the agar disc diffusion method. Four Gram positive (*Staphylococcus aureus*, *Micrococcus* sp., *Bacillus subtilis* and *Bacillus thuringensis*), four Gram negative (*Escherichia coli*, *Klebsiella pneumonia*, *Salmonella typhi* and *P. mirabilis*) and one yeast (*Candida albicans*) species were screened. Inhibition zones ranged between 16–29 mm. Leaves extract inhibited the growth of all tested microorganisms with large zones of inhibition, followed by that of flowers, which also inhibited all the bacteria except *C. albicans*. The most susceptible microbes to all extracts were *S. aureus* and *Micrococcus* sp. Root extract displayed larger inhibition zones against Gram positive bacteria than Gram negative bacteria and had larger inhibition zones compared to stem extract. The lowest MIC values were obtained with *E. coli* and *C. albicans* (3.12 mg/mL), followed by *S. aureus* (12.50 mg/mL) and *P. mirabilis* (50.00 mg/mL). All the other bacteria had MIC values of 100.00 mg/mL. Scanning Electron Microscopic (SEM) studies revealed that the cells exposed to leaf extract displayed a rough surface with multiple blends and invaginations which increased with increasing time of treatment, and cells exposed to leaf extract for 36 h showed the most damage, with abundant surface cracks which may be related to final cell collapse and loss of function. Time-kill assay of *C. albicans* indicated a primarily fungicidal effect at 1- and 2-fold MIC. *E. hirta* extracts had LC_50_ values of 0.71, 0.66, 0.41 and 0.03 mg/mL for stems, leaves, roots and flowers, respectively against *Artemia salina*. Hence, these plants can be used to discover new bioactive natural products that may serve as leads in the development of new pharmaceuticals.

## 1. Introduction

Despite the huge number of antimicrobial agents for various purposes that already exist, the search for new drugs is a continuous task since the target microorganisms often develop new genetic variants which subsequently become resistant to available antimicrobial agents and the effective lifespan of any antibiotic is thus limited. The world’s attention is now increasingly directed towards plant sources for developing antimicrobial drugs, since natural products are considered safer than synthetic ones. According to the World Health Organization, medicinal plants would be the best source to obtain a variety of drugs. Therefore, such plants should be investigated to better understand their properties, safety and efficacy [1]. There are several published reports describing the antimicrobial activity of various crude plant extracts [2,3]. It is estimated that there are about 2.5 million species of higher plants and the majority of these have not yet been examined for their pharmacological activities [4].

*Euphorbia hirta* L. belongs to the family Euphorbiaceae. It is a small annual herb common to tropical countries. It is usually erect, slender-stemmed; spreading up to 80 cm tall, though sometimes it can be seen lying down. The plant is an annual broad-leaved herb that has a hairy stem with many branches from the base to the top. The leaves are opposite, elliptical, oblong or oblong-lanceolate, with a faintly toothed margin and darker on the upper surface. The flowers are small, numerous and crowded together in dense cymes (dense clusters in upper axils) about 1 cm in diameter. The stem and leaves produce a white or milky juice when cut. It is frequently seen occupying open waste spaces, banks of watercourses, grasslands, road sides, and pathways [5,6].

*E. hirta* is a very popular herb amongst practitioners of traditional medicine, widely used as a decoction or infusion to treat various ailments including intestinal parasites, diarrhoea, peptic ulcers, heartburn, vomiting, amoebic dysentery, asthma, bronchitis, hay fever, laryngeal spasms, emphysema, coughs, colds, kidney stones, menstrual problems, sterility and venereal diseases. Moreover, the plant is also used to treat affections of the skin and mucous membranes, including warts, scabies, tinea, thrush, aphthae, fungal afflictions, measles, Guinea-worm and as an antiseptic to treat wounds, sores and conjunctivitis. The plant has a reputation as an analgesic to treat severe headache, toothache, rheumatism, colic and pains during pregnancy. It is used as an antidote and pain relief of scorpion stings and snakebites. The use of the latex to facilitate removal of thorns from the skin is common [7]. The sedative, anxiolytic, analgesic, antipyretic and anti-inflammatory properties of *E. hirta* have been reported in the literature [8]. Leaf extract of *E. hirta* increased urine output and electrolytes in rats [9]. Furthermore, studies revealed that *E. hirta* posses galactogenic, anti-anaphylactic, antimicrobial, antioxidant, anticancer, antifeedant, anti-platelet aggregation and anti-inflammatory, aflatoxin inhibition, antifertility, anthelmintic, antiplasmodial, antiamoebic, antimalarial, larvicidal, and repellent and antifeedant activities against *Plutella xylostella* [6].

Although, many studies have been done on *E. hirta,* none of them were performed on the separated parts of the plant, as most studies one involved the whole plant or leaves. The objective of this study was to evaluate the potential antimicrobial activity of *E. hirta* leaves, flower, stem and root extracts against common pathogenic bacteria and fungi. The antifungal activity of the leave extract against *C. albicans* was studied in detail, starting from disc diffusion (zone of inhibition) and broth dilution (MIC and MFC) to time kill and scanning electron microscopic studies. Moreover, *E. hirta* extracts’ toxicity was evaluated using brine shrimp lethality assay. TEM and oral acute toxicity studies are ongoing. 

## 2. Results and Discussion

### 2.1. Disc diffusion assay

The observed antimicrobial activity of *E. hirta* expressed as zone of inhibition (mm) is shown in Table 1. Methanolic extract (100.00 mg/mL) of all the parts displayed good antibacterial activity against Gram positive (*Microccus* sp., *Staphylococcus aureus*, *Bacillus subtilis*, and *Bacillus thuringiensis*), Gram negative (*Escherchia coli*, *Salmonella typhi, Klebsiella pneumonia* and *Proteus mirabilis*) bacteria and the fungus *C. albicans*. Inhibition zones ranged from 16–29 mm. Leaves extract exhibited the best activity, since it inhibited the growth of all tested microorganisms with large zones of inhibition, followed by flowers, which also inhibited all the bacteria except the yeast *C. albicans* which was inhibited only by the leaves. *S. aureus* and *Micrococcus* sp. were, in general, the most susceptible microbes to all extracts. Only half the bacteria (three Gram positive and one Gram negative species) were inhibited by the stem and root extracts. Noticeably, what was inhibited by stems was also inhibited by roots and the opposite is also true, as resistant bacteria were so with both extracts. However, root extract displayed larger inhibition zones against Gram positive bacteria than Gram negatives and had larger inhibition zones compared to stem extract. The standard antibiotics chloramphenicol and miconazole nitrate were found to have zone of inhibitions 21–26 mm at the concentration of 30 µg/disc. In contrast, the inhibition zone of methanol (negative control) was almost zero for all the tested microorganisms.

The large inhibition zones exhibited by the extract against *S. aureus* justified the plant use in the treatment of sores, bores and open wounds. Gram-negative bacteria showed weaker inhibition zones than Gram positive ones, which may be related to the presence of a thick phospholipid and lipopolysaccharide outer membrane layer that protects them from environmental factors and makes them highly resistant, even to synthetic antibiotics [10]. Differences in the plant parts’ activities may be due to the various bioactive compounds available in each part. Resistant microorganisms do not indicate the absence of bioactive constituents, nor that the plant is inactive, rather, active compound(s) could be found in insufficient quantities in the crude extracts to show activity at the tested concentrations. Lack of activity can thus only be proven by using large doses [11]. On the other hand, there could also be other constituents exerting antagonistic effects on the bioactive compounds or the method used for extraction couldn’t extract all the bioactive compounds. For example, polar and non polar solvents extract different compounds according to their respective solubility in these solvents. However, to know the exact mechanism of action of the extracts, further studies with purified fractions, different solvents and various methods of extractions (hot/cold, acidic/basic, fresh/dry plant, *etc.*) are suggested to reveal the actual antimicrobial activities.

**Table 1:** Antimicrobial activity of *Euphorbia hirta* expressed as zone of inhibition (mm).

Microorganism	Zone of Inhibition (mm)					
	Leaves	Stems	Flowers	Roots	Chloramphenicol	Miconazole nitrate
*Staphylococcus aureus* (+)	28	16	28	21	23	ND
*Bacillus thuringiensis* (+)	21	17	15	20	25	ND
*Bacillus subtilis* (+)	16	R	15	R	23	ND
*Micrococcus sp.* (+)	29	15	28	19	26	ND
*Escherichia coli* (-)	18	R	15	R	24	ND
*Klebsiella pneumonia* (-)	19	R	12	R	24	ND
*Proteous mirabilis* (-)	19	17	9	15	23	ND
*Salmonella typhi* (-)	18	R	16	R	21	ND
*Candida albicans* (fungus)	21	R	R	R	ND	21

(+): Gram positive bacteria, (-): Gram negative bacteria, R: Resistant, ND: Not determined. The inhibition zone diameter was taken as an average value of triplicate plates for each microorganism at 25 µL of 100 mg/mL crude extract, 30 µg/mL of chloramphenicol and 30 µg/mL of miconazole nitrate.

### 2.2. Minimum inhibitory and fungicidal concentrations

The minimum inhibitory concentrations of the leaf extract on the test isolates are shown in Table 2. The MIC values ranged from 3.13–100 mg/mL. The lowest MICs were against *E. coli* and *C. albicans*, with a concentration of 3.13 mg/mL, followed by *S. aureus* and *P. mirabilis* with values of 12.50 and 50.00 mg/mL, respectively. Furthermore, the extract had a MIC of 100.00 mg/mL against *B. subtilis, B. thuringensis, Micrococcus* sp., *K. pneumonia* and *S. typhi.* Earlier studies reported that the MBC values can either be the same or higher than the corresponding MIC values [10], but in this study, the MIC were the same as the MBC values. Consequently, the MBC values which are obtained after plating various dilutions of the extracts are more reliable than the MIC values, obtained using turbidity as a measure of growth. 

Lower MIC and MBC values indicate higher efficacy [12]. Thus, the low MBC values exhibited by the leaf extract against *E. coli* and *C. albicans* is of potential importance in the health care delivery system, since it could be used as an alternative to orthodox antibiotics in the treatment of infectious diseases caused by these microbes, especially as they frequently develop resistance to known antibiotics [13].

**Table 2:** Minimal Inhibitory and Bactericidal Concentration of *Euphorbia hirta* leave extract.

Microorganism	MIC (mg/mL)	MBC&MFC (mg/mL)
*Staphylococcus aureus* (+)	12.50	12.50
*Bacillus thuringiensis* (+)	100.00	100.00
*Bacillus subtilis* (+)	100.00	100.00
*Micrococcus sp.* (+)	100.00	100.00
*Escherichia coli* (-)	3.13	3.13
*Klebsiella pneumonia* (-)	100.00	100.00
*Proteous mirabilis* (-)	50.00	50.00
*Salmonella typhi* (-)	100.00	100.00
*Candida albicans* (Fungus) MFC	3.13	3.13

MIC: Minimal Inhibitory Concentration; MBC: Minimal Bactericidal Concentration; MFC: Minimal Fungicidal Concentration.

### 2.3. Time kill study

**Figure 1:**
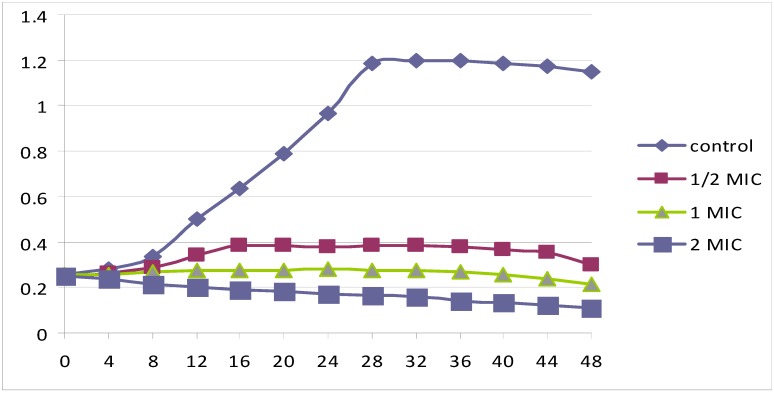
Growth curves of *Candida albicans* in Muller Hinton broth with 0 (control), ½, 1 and 2 times MIC (3.125) of *Euphorbia hirta* leaves extract after 48 h incubation.

The growth profile curves in Figure 1 demonstrate that *Euphorbia hirt* leaves extract was fungicidal against *C. albicans.* The ½ MIC curve exhibited a similar shape as the control curve, but with a down shift for which the OD values were always higher. Each of the two higher concentrations (1 and 2 MIC-folds) exhibited fungicidal activity during the 48 h duration of the test, with a great drop of OD compared to the control and starting inoculums. No recovery in *C. albicans* cells growth was seen throughout the experiment. These observations of the present study correlate with the anti candidal activities displayed by disc diffusion and broth dilution methods and demonstrate the extract’s potent anti-candidal activity.

### 2.4. Scanning electron microscope (SEM) observations

**Figure 2:**
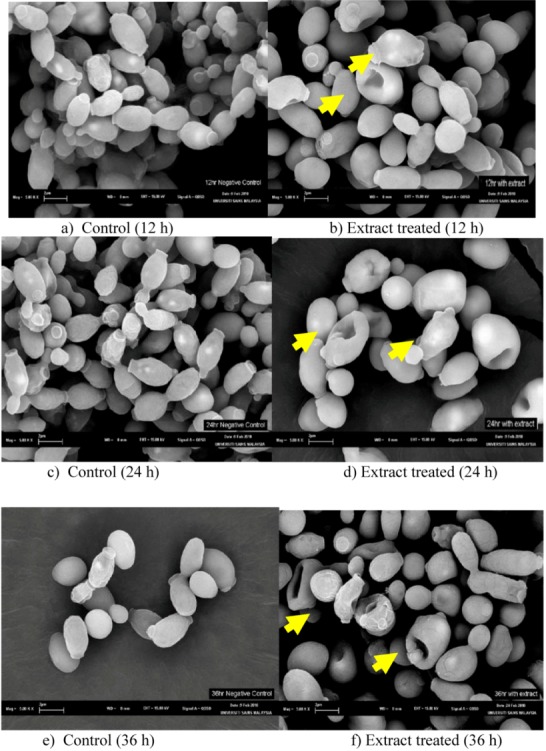
Scanning electron microscopic images (5.00 k x) of *Candida albicans* cells before and after treating with *Euphorbia hirta* leaves extract for 12, 24 and 36 h.

By SEM all the control cells of *C. albicans* after 12, 24 and 36 h were generally smooth-walled bodies, spherical to elongate in shape and were mostly present in yeast form (Figure 2). All the yeast cells were lying apart, showing polar buds and bud scars after 12 h treatment with *E. hirta* leaves extract, and several small blebs appeared on some of the cell surfaces. The remaining cells showed a smooth surface, as observed in control cells. More invaginations and convolutions (arrows) appeared in the 24 h treated cells. Cracks in the cell wall were only detected in the last sample, which was treated with the leaf extract for the longest duration (36 h). Thus, it is assumed that at this stage, the cells had lost its metabolic functions.

The SEM observations presented in this study clearly confirm the potent fungicidal activity exerted by the leaf extract. The surface alterations are most probably due to a change in cell permeability, which is in agreement with earlier ultrastructural observations, showing that the first changes are localized at the plasmalemma and cell wall with progressive cytoplasmic deterioration and prominent shape changes, before any alteration can be detected in the cell interior end with complete cell necrosis [14].

### 2.5. Brine shrimp lethality bioassay

The LC_50_ value of methanol extracts is shown in Table 3, the crude methanol extract of *E. hirta* parts showed positive results, indicating that it is biologically active. The mortality rates of brine shrimp were found to be increased with increasing sample concentrations. 

**Table 3:** LC_50_ of methanolic extract of *Euphorbia hirta.*

Sample	LC_50_ value
Potassium dichromate	0.076
Stem extract	0.710
Leaves extract	0.660
Root extract	0.413
Flower extract	0.033

Values are expressed as an average of triplicates.

The results on brine shrimps assay indicate that all parts of *E. hirta* except the flower extract had LC_50_ values less than 1,000 μg/mL. This suggests that *E. hirta* leaves, stems and roots might be toxic to humans, so caution must be taken when consuming the plant and a safe dose must be considered. The disc diffusion assay revealed that *E. hirta* extracts showed a significant antimicrobial activity against the screened strains, so the toxic properties of the plant do not mean that use of *E. hirta* as an antimicrobial agent should stop; on the contrary, if the active ingredients can be isolated and identified, it maight be used in the synthesis of useful pharmaceuticals such as strong antiseptic agents which are crucial means of maintaining hygienic conditions. TEM and oral acute toxicity studies are also ongoing to confirm the SEM and time kill assay exerted by the leaves extract against *C. albicans.*

## 3. Experimental

### 3.1. Plant collection

The fresh plant leaves was harvested from various areas in Universiti Sains Malaysia, Penang, Malaysia in June 2009. The taxonomic identity of the plant was confirmed by the botanist of the School of Biological Sciences at Universiti Sains Malaysia (voucher number 11077). The plant materials were washed under tap water and separated into leaves, flowers, stems and roots. The separated parts were air dried in shade for ten days and then in an oven at 60 ºC for one to two days, before grinding to a fine powder using an electric blender and stored in clean labeled airtight bottles. 

### 3.2. Preparation of the plant extract

A hundred grams of powder of each part was extracted by maceration in methanol (400 mL) for 14 days with frequent agitation. The mixture was filtered through clean muslin cloth followed by double filtration with Whatman No.1 filter paper and the filtrate was concentrated by rotary evaporation under vacuum (vacuum pressure: 500 N/m^2^) at 40 ºC until a volume of about 15 mL wast reached. Next the concentrate was poured into glass Petri dishes and brought to dryness in an oven at 60 ºC. The percentage yield of the crude extract was determined for each part and was 11.1%, 7.3%, 4.7% and 4.1% for leaves, stems, flowers and roots, respectively. The obtained paste like mass was then stored in parafilm sealed Petri-dishes in a dark cabinet. The extracts were reconstituted by dissolving in methanol to the required concentrations. The reconstituted extracts were maintained at 2–8 ºC.

### 3.3. Test microorganisms and growth media

Four Gram positive (*Staphylococcus aureus*, *Micrococcus* sp., *Bacillus subtilis* and *Bacillus thuringensis*), four Gram negative (*Escherichia coli*, *Klebsiella pneumonia*, *Salmonella typhi* and *Pseudomonas aeruginosa*) and one yeast (*C. albicans*) species were obtained from the Microbiology Laboratory stocks, School of Biological Sciences. The bacterial strains were grown in nutrient agar (NA) plates at 37 ºC, whereas the yeast was grown in Sabouraud dextrose agar (SDA) at 28 ºC. The stock cultures were maintained at 4 ºC. 

### 3.4. Determination of the antimicrobial activity

#### 3.4.1. Disc diffusion assay

Antibacterial and antifungal activities of plant parts extracts were investigated by the disc diffusion method [15]. The Müller-Hinton agar (MHA) plates, containing an inoculum size of 10^6^ colony-forming units (CFU)/mL of bacteria or 2 × 10^5^ CFU/mL yeast cells on SDA, were spread with a sterile cotton swab. Then discs (6.0-mm diameter) impregnated with 25 μL of each extract at a concentration of 100.0 mg/mL were placed on the inoculated plates. Similarly, a disc with 25 μL methanol served as negative control, 30 μg/mL chloramphenicol, and 30 μg/mL miconazole nitrate were also used as positive controls for bacteria and yeast, respectively. The plates were allowed to stand for 30 min for prediffusion of the extract to occur and then incubated at 37 ºC for 18 to 24 h for bacteria and at 28 ºC for 48 h for yeast and the zones of inhibition (including the diameter of disk) were measured to the nearest mm. The mean of triplicate results was calculated.

#### 3.4.2. Determination of minimum inhibitory and fungicidal concentrations

Determination of the minimum inhibitory concentration (MIC) was carried out only on leaves extract using the twofold broth dilution method [10] with some modifications. Briefly, the reconstituted extract solution (2.0 mL) at a concentration of 200.00 mg/mL was added to another test tube containing sterile broth (2 mL) so as to obtain a concentration of 100.00 mg/mL. Two-fold serial dilution of the extract was made from the initial concentration of 100.00 mg/mL. One tube without extract was used as negative control. Then an 18 h old culture (0.5 mL) of each of the bacteria earlier adjusted to 0.5 Mc Farland (10^6^ CFU/mL) was added into each tube and mixed well by the micropipette. The tubes were loosely closed and incubated at 37 ºC for 24 h and then observed for growth in form of turbidity. The test was performed in triplicate for each bacterium. The test tube with the lowest dilution with no detectable growth by naked eye was considered the MIC. The same procedure was used for *C. albicans* but with changing the inoculums with 0.5 mL of 0.5 Mc Farland standard *C. albicans*. All the MIC tubes, which did not show any turbidity, were streaked over the Muller–Hinton agar plates for bacteria and Sabouraud’s dextrose agar plates for *C. albicans* and incubated at 37 ºC for 24 h. The minimum bactericidal/fungicidal concentration was recorded as the lowest concentration that did not permit any visible growth on the plates after the period of incubation

### 3.5. Time kill study

*E. hirta* leaves extract was evaluated for the anti-candidal effect with ½, 1 and 2 times MIC over 48 h and the growth profile curve was plotted [16]. A 16 hrs culture was harvested by centrifugation, washed twice with sterile phosphate saline buffer (SPSB) and resuspended in SPSB. The suspension was then adjusted using McFarland standard and further diluted in SPSB to obtain approximately 10^6^ CFU/mL. Reconstituted leaves extract was added to 25 mL Muller Hinton broth (MHB) tubes to achieve a final concentration of ½, 1 and 2 times MIC value (3.12 mg/mL). One mL of previously prepared inoculum was added to each solution tube. Extract-free medium served as a control. Then 1 mL was aseptically removed from each tube and the growth of *C. albicans* was monitored at predetermined time points at 4 fold time series during 48 h by measuring the optical density (OD) at 540 nm. All solutions were incubated in 37 ºC water bath. The growth profile curve was plotted using Microsoft Excel.

### 3.6. Scanning electron microscope observations:

Only leaves extract was used for *C. albicans* scanning electron microscope observations [16]. Six SDA plates were inoculated with *C. albicans* and incubated at 30 ºC for 6 h. One mL of 3.13 mg/mL leaves extract was added to each of a three of the inoculated SDA plates and incubated at the same temperature. Methanol treated plates served as controls. The scratching method was used to harvest the *C. albicans* cells from the plates at 12, 24, and 36 h and samples were then fixed in McDowell-Trump fixative prepared in 0.1 M phosphate buffer for 24 h and processed for scanning. Processed samples were desiccated and coated with gold before were viewed under SEM (LEO SUPRA 50 VP Feild Emission SEM, Germany). The same was done for the controls. 

### 3.7. Brine shrimp lethality bioassay

The experiment was carried out using the method described by Meyer. Briefly, *Artemia salina* cysts (brine shrimp eggs. 0.1 g) were allowed to hatch and mature as nauplii i and ii in filtered artificial seawater (100 mL, 38% w/v salt in distilled water) for 48 h at 25 ºC under constant aeration and illumination. Ten-fifteen nauplii were collected with a pipette from the lighted side and added to the two-fold serially diluted test solutions (100.00–0.19 mg/mL extract in 4 mL artificial seawater). Potassium dichromate (1000.00–1.95 µg/mL) served as positive control. After the 24 h incubation at 25 ºC, a magnifying lens used to count the number of dead and the mortality percentage was calculated. Larvae were considered dead only if they did not move for few seconds during observation. The triplicate mean of percentage mortality was plotted against the concentrations logarithm using Microsoft Excel. Equation and regression appeared on the graph, so LC_50_ was calculated from the linear equation by taking the antilogarithm. Extract was considered bioactive if the LC_50_ was less than 1 mg/mL [17].

## 4. Conclusions

Bacterial and Candidal infections can be treated with the *E. hirta* extract, since it exhibited favorable antibacterial and anticandidal activities, but it should be noticed that the plant should be consumed with small doses since it is found to have toxic effects in a brine shrimp assay. The separated parts of *E. hirta* have never been evaluated for antibacterial and anticandidal activity before. Therefore, it is the first time they have been studied separately and in detail using a broad range of microbial samples. On the basis of the present study, further phytochemical and pharmacological studies will be needed to isolate the bioactive compound(s) and investigate the antimicrobial activities against a wider range of pathogenic microorganisms. 
